# Antenatal care use in urban areas in two European countries: Predisposing, enabling and pregnancy-related determinants in Belgium and the Netherlands

**DOI:** 10.1186/s12913-016-1478-3

**Published:** 2016-08-02

**Authors:** Jana Vanden Broeck, Esther Feijen-de Jong, Trudy Klomp, Koen Putman, Katrien Beeckman

**Affiliations:** 1 Organisation, Policy and social Inequalities in Health care (OPIH), Department of Medical Sociology and Health Sciences, Vrije Universiteit Brussel, Brussels, Belgium; 2 Department of Nursing and Midwifery, Nursing and Midwifery Research Unit, University Hospital Brussel, Laarbeeklaan 101, 1090 Brussels, Belgium; 3 Department of Midwifery Science, AVAG, Groningen and EMGO Institute for Health and Care Research, VU University Medical Center, Amsterdam, The Netherlands; 4 Interuniversity Centre for Health Economics Research, Vrije Universiteit Brussel, Brussels, Belgium

## Abstract

**Background:**

Examining determinants of antenatal care (ANC) is important to stimulate equitable distribution of ANC across Europe. This study (1) compares ANC utilisation in Belgium and the Netherlands and (2) identifies predisposing, enabling and pregnancy-related determinants.

**Methods:**

Secondary data analysis is performed using data from Belgium, and the Netherlands. The content and timing of care during pregnancy (CTP) tool measured ANC use. Non-parametric tests and ordinal logistic regression are performed to gain insight in the determinants of health care use.

**Results:**

Dutch women receive appropriate ANC more often than Belgian women. Multivariate analysis showed that lower education, unemployment, lower continuity of care and non-attendance of antenatal classes are associated with a lower likelihood of having more appropriate ANC.

**Conclusions:**

Predisposing and pregnancy related variables are most important to influence the content and timing of ANC, irrespective of the country women live in. Lower health literacy in socially vulnerable women might explain the predisposing determinants of health care use in both countries. Stimulating accessibility to antenatal courses or organising public education are recommendations for practice. Regarding pregnancy-related determinants, improving continuity of care can optimise ANC use in both countries.

## Background

An understanding of the individual determinants (patient-related factors) of antenatal care (ANC) utilisation may assist the pursuit of adequate levels of care recommended for every pregnancy. ANC is important because it enables early and continuing risk assessment, health promotion and medical and psychosocial follow-up [1]. Despite its value, some women do not make proper use of ANC [2].

According to Andersen and Newman ‘s health behavioural model, individual determinants of health care utilisation can be divided into predisposing, enabling [3] and need components [4]. With respect to ANC, predisposing determinants refer to individual characteristics which exist prior to the pregnancy and affect the propensity to use care. Previous studies have concluded that low maternal age [4–7], being single [7], low educational level [6–9], lack of a paid job [9], foreign ethnic background [6, 9] or origin [2, 5, 8], poor language proficiency [1, 7], (little) support from a social network [1] and lack of knowledge of the health care system [1] are associated with inadequate ANC utilisation. Enabling determinants refer to conditions which make ANC available to pregnant women. Absence of health insurance [6, 7], planned pattern of ANC [6], hospital type at booking [6], personalized communication and knowledge of cultural practices of the care provider [1] have been found to be associated with inadequate ANC. The pregnancy-need component of the determinants include pregnancy related elements explaining the degree of care needed/used. Inadequate use of ANC seems to be related to high parity [5–7], unplanned pregnancy [7], no previous premature birth [6], discontinuity of care [8], late recognition of pregnancy [6] and behavioral factors such as smoking during pregnancy [6, 9].

The measurement of ANC utilisation varies across studies, therefore results must be interpreted cautiously. The initiation of care [1, 5–7, 9], the number of antenatal visits [6, 7] and several indices based on the timing of initiation of ANC, the total number of antenatal visits and the gestational age at birth [2, 6–8] have been used previously to define ANC use. Since there is no consensus about the number of antenatal visits [10], it is preferable to take into account elements of the content and timing of care during the pregnancy. One recent study measured ANC more comprehensively using the content and timing of care during pregnancy (CTP) tool [8].

Previously defined determinants of ANC use should be interpreted in relation to the context of these studies. In addition to individual determinants, health care utilisation depends on resources (e.g. number of care providers available) and the organisation of the national health care system, such as the nature of referrals between health care providers [3]. Feijen-de Jong et al. identified the need for comparative research in several countries with varying antenatal health care arrangements as these might explain differences in the effects of individual determinants on ANC use [6]. Therefore in this study, we compared ANC between two groups of ANC attendees in two different countries (Belgium and the Netherlands) with a different health care system. In the Netherlands, most women with uncomplicated pregnancies receive ANC from primary care midwives who act as gatekeepers to secondary obstetric care [11]. They receive fixed remunerations for follow-up during the full length or part of the pregnancy [12]. In Belgium, most women access an obstetrician directly for ANC as they do not need preauthorisation to gain access to specialist care [5]. The majority of general practitioners, specialists and independent midwives in Belgium are paid on a fee-for-service basis [13].

This study aims to 1) compare ANC utilisation in Belgium and the Netherlands as measured by the CTP tool and 2) to identify its predisposing, enabling and pregnancy-related determinants.

## Methods

### Data collection

A secondary data analysis is performed using pooled data from two studies. For Belgium, data were obtained from a prospective observational study conducted in the Brussels Metropolitan Region (the CTP study) [10]. Recruitment occurred between April and July 2008 in nine out of 12 hospital centres for ultrasound to which every woman is referred. All low risk women, at the beginning of their care trajectory (attending a first or second visit or having a gestational age less than 16 weeks) were elegible for inclusion. Data collection comprised a questionnaire about personal characteristics and pregnancy history at the moment of recruitment, a diary recording all antenatal visits in a structured manner (for each visits, 6 questions needed to be filled out, for each question closed answers were provided, women needed to copy the code related to their answer) and bimonthly (once in two months) telephone follow-up interviews to record ANC use (*n* = 333) [10]. This study was approved by all participating centers and from the Ethics Committee of the University Hospital UZ Brussel.

For the Netherlands, data were obtained from the DELIVER (Dutch acronym for ‘data primary care delivery’) study. Data were gathered in a 12 month study period in 2009-2010. The Deliver study is a descriptive study that aimed to provide information about midwifery care organization, accessibility of midwifery care, and the quality of primary midwifery care in the Netherlands [14]. Midwifery practices were recruited by using purposive sampling. Three stratification criteria were used: region (north, east, south, west), level of urbanisation (urban or rural area), and practice type (dual or group practice) to ensure that different types of practices in different regions were represented. Subsequently, all clients receiving care in the participating primary midwifery practices at any moment in a 12 month study period in 2009–2010 were eligible to participate if they were able to understand Dutch, English, Turkish or Arabic. The participating practices (20 of the 519 midwifery practices in the Netherlands) comprised 110 midwives and a caseload of 8200 clients per year, with all regions of the Netherlands being represented [14]. Data collection with regard to pregnant women recruited in primary care midwifery practices included up to two questionnaires about socio-demographic characteristics and ultrasound scans. One questionnaire was administered before 34 weeks of gestation and the other between 34 weeks of gestation and birth. In addition, information about antenatal care utilization was gathered by extracting data from electronic client records of participating clients. This study was approved by the Medical Ethics Committee of the VU University Medical Center Amsterdam.More study details can be found in the specific papers [10, 14].

### Composition of the pooled data set

**Fig. 1:**
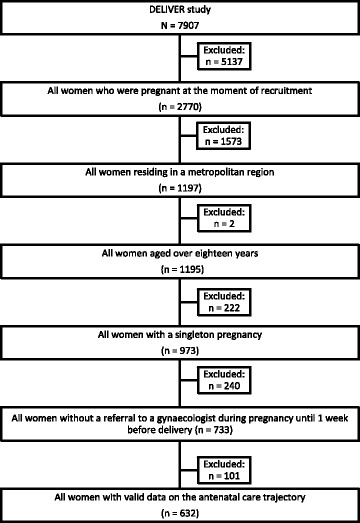
Overview of the selection of the DELIVER subsample applying the common inclusion criteria

To have comparable inclusion criteria for the secondary data analysis, only adult women (>18 years) residing in an urban region (2500 or more households per km^2^) with a low-risk onset of pregnancy (without pre-existing medical complications) were eligible for inclusion. Application of these criteria meant a reduction of the Dutch study sample to 632 women (Fig. 1). Because of the unbalance in the numbers between both samples, a pooled data set was constructed by combining the entire sample from the Belgian study (*n* = 333) and a random matched sample from the 632 women remaining in the Dutch study.

**Fig. 2:**
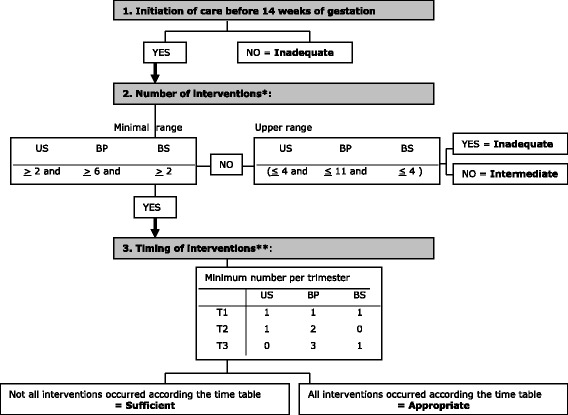
outline of the Content and Timing of care in Pregnancy (CTP) tool ^10^. US: Ultrasound, BP: Blood Pressure, BS: Blood Screening, T: Trimester. *Ranges based on the NICE and Belgian guideline. **Ranges based on the NICE guidelines. *Inadequate*: initiation of care after first trimester OR the number of at least one intervention is less than the lower range and none of the interventions occurred more than the range. *Intermediate*: initiation of care in the first trimester; the number of at least one intervention occurred less than the lower range and at least one intervention exceeded the range. *Sufficient*: initiation of care in the first trimester; the number of all interventions equals at least the respective lower range but timing of at least one intervention is not as recommended. *Appropriate*: initiation of care in the first trimester; the number of the interventions equals at least the respective lower range and timing of the actions of all basic interventions is as recommended

To reduce possible pre-existing differences in distribution between both populations, predictors for antenatal care use were used to define a comparable dataset. Our first step was to reduce missings in the Dutch data. Multiple imputation was performed for missing values with regard to household income (97/632), using the hot deck method [15]. Seen Chi-square analyses indicated that non-response concerning household income depends on a woman’s occupational status and educational level (*p* < 0.05). Missing values of non-respondents were replaced by observed values from a respondent similar to the non-respondent [16] for these variables. Five imputed data sets were generated to calculate the mean household income for each non-respondent. There were no missings in the Belgian study.

After completing the imputation in the Dutch sample we observed that the individual characteristics of both subsamples were distributed differently. Women in the Belgian subsample were significantly (*p* < 0.05) younger (aged ≤ 20), were more often single, more often less educated, less likely to be active on the labour market and were more often of a foreign nationality compared to the Dutch subsample. In the Belgian sample, women had more often a less educated partner (*p* < 0.05) and more often a partner with a foreign nationality. Furthermore, these women more often had a low and high equivalent income (*p* < 0.05) and lack of health insurance and additional health insurance cover. Finally, these women were more often multiparae (*p* < 0.05), had more unwanted pregnancies, more unplanned pregnancies and attended fewer antenatal information classes. These observed differences might potentially influence differences in health care utilisation, therefore exact matching without replacement [17] was conducted in order to balance the distribution of individual characteristics between the subsamples of the pooled data set. The units of the Dutch subsample were ordered at random and were matched 1:1 to the units of the Belgian subsample for two variables: educational level [6, 7, 9] and maternal age [5–7]. These variables were chosen because in literature they were observed to be predisposing determinants of ANC use. For 321 women in the Belgian sample we were able to match with someone in the Dutch study. The final pooled dataset (*n* = 642) therefore consisted of 321 women from Belgium and 321 from the Netherlands.

### Operationalization of ANC utilisation by the CTP tool

The CTP tool (Fig. 2) considers three dimensions: the timing of initiation of care, and the number and timing of three specific interventions during pregnancy (blood screening, ultrasound and blood pressure measurement) [10]. Four categories of ANC use are defined by the CTP: inadequate, intermediate, sufficient or appropriate care. This classification reflects the degree to which a minimum amount of care recommended by national obstetric guidelines for every pregnancy was received, regardless of parity or risk status [10]. As the CTP was developed based on evidence about the importance of interventions in pregnancy and the congruence of ANC guidelines, the tool is applicable in the Netherlands [18–21].

### Potential individual determinants of ANC utilisation

The original data collection instruments used in both studies were explored to determine the variables that had been equivalently operationalised. The common variables to form the predisposing component were age, marital status, educational level, occupational status and current nationality. In addition, educational level and current nationality of the partner were examined. A variable for region referred to the two original study samples: the Metropolitan Region of Brussels, Belgium and urban regions in the Netherlands. The educational level of all women was classified into three categories according to the International Standard Classification of Education (ISCED) [22].

The variables reflecting the enabling component were equivalent income, health insurance cover and additional health insurance cover. Equivalent income was calculated by using the modified Organization for Economic Co-operation and Development (OECD) scale and classified into three categories. This scale involves adjusting monthly household income based on its size and the age of its members [23]. The lowest income group was defined at < 60 % of the respective median national income [24], the at-risk-of-poverty threshold [25]. The moderate and high income groups were delineated at 60–120 % and > 120 % of the national median equivalent net income respectively.

The variables describing the pregnancy-related component were parity, wish for pregnancy, planned pregnancy, continuity of care and attendance of antenatal information classes. Continuity of care was measured by the Continuity of Carer (COC) index, based on the number of visits to each different health care provider and the total number of visits [26]. The index, expressed in percentage, was divided into two categories, with the cut-off point < 50 % and ≥ 50 %.

### Statistical analysis

For each region, the individual characteristics of the study sample and ANC utilisation were summarised. Individual characteristics and ANC utilisation were compared between regions using Chi-squared tests, the association between each of the individual characteristics and ANC utilisation for the whole sample was determined (Chi-squared tests). Subsequently, logistic ordinal regression analysis was used to examine the significance of each individual characteristic in terms of its likelihood of being given a higher CTP classification, while controlling for the remaining significant characteristics. Since this was an exploratory study, backward elimination was used (stay level: *p* < 0.05) [27]. Our model was constructed in three steps in accordance with the health behaviour model [3, 4]. The first step considered predisposing variables, the second step considered enabling factors, with the selected predisposing variables fixed in the model, and in the final step the pregnancy-related variables were examined while controlling for the selected predisposing and enabling variables. In order to include other variations between the subsamples, the variable region was fixed in this model from the first step onwards. A Score test for the proportional odds assumption and absence of multicollinearity was undertaken for each step. In addition, the final model assessed the percentage of concordant pairs of predicted probabilities and observed responses (>60 %). Multivariate analyses were conducted in SAS 9.1, and all other analyses were performed using SPSS Statistics 20.

## Results

### Characteristics of the women

The final data set consisted of 642 women. Chi-squared tests indicated significant differences between the two subsamples for marital status, occupational status, nationality, educational level of the partner, nationality of the partner, equivalent income, health insurance or additional health insurance cover, parity, desire for pregnancy and attendance of antenatal information classes (*p* < 0.05) (Table 1).

**Table 1:** Study sample characteristics, comparison between both groups of antenatal care attendees (*n* = 642)

	Total	Brussels Metropolitan Region	The Netherlands	
	(*N* = 642)	(*N* = 321)	(*N* = 321)	Chi^2^
				(*p*-value)
	N (row %)	N (column %)	N (column %)	
Predisposing characteristics				
Age (years)				1.000
< =20	8 (1.2)	4 (1.2)	4 (1.2)	
21-35	528 (82.2)	264 (82.2)	264 (82.2)	
> 35	106 (16.5)	53 (16.5)	53 (16.5)	
Marital status				**.000**
Co-habiting or married	604 (94.1)	291 (90.7)	313 (97.5)	
Single	38 (5.9)	30 (9.3)	8 (2.5)	
Educational level				1.000
Up to secondary	376 (58.6)	188 (58.6)	188 (58.6)	
Tertiary	266 (41.4)	133 (41.4)	133 (41.4)	
Occupational status				**.000**
Employed	419 (65.3)	149 (46.4)	270 (84.1)	
Unemployed	223 (34.7)	172 (53.6)	51 (15.9)	
Nationality				**.000**
Belgian/Dutch	475 (74.0)	184 (57.3)	291 (90.7)	
All other nationalities	167 (26.0)	137 (42.7)	30 (9.3)	
Educational level partner				.001
No partner	38 (5.9)	30 (9.3)	8 (2.5)	
Up to secondary	334 (52.0)	163 (50.8)	171 (53.3)	
Tertiary	270 (42.1)	128 (39.9)	142 (44.2)	
Nationality of the partner				**.000**
No partner	38 (5.9)	30 (9.3)	8 (2.5)	
Belgian/Dutch	441 (68.7)	170 (53.0)	271 (84.4)	
All other nationalities	163 (25.4)	121 (37.7)	42 (13.1)	
Enabling characteristics				
Equivalent income^a^				**.000**
Low	112 (17.4)	92 (28.7)	20 (6.2)	
Moderate	451 (70.2)	151 (47.0)	300 (93.5)	
High	79 (12.3)	78 (24.3)	1 (0.3)	
Health insurance coverage				**.000**
Yes	623 (97.0)	302 (94.1)	321 (100.0)	
No	19 (3.0)	19 (5.9)	0 (0.0)	
Additional health insurance coverage				**.000**
Yes	431 (67.1)	151 (47.0)	280 (87.2)	
No	211 (32.9)	170 (53.0)	41 (12.8)	
Pregnancy-related characteristics				
Parity				**.001**
Primiparae	284 (44.2)	121 (37.7)	163 (50.8)	
Multiparae	358 (55.8)	200 (62.3)	158 (49.2)	
Wish for pregnancy^b^				**.002**
Wanted pregnancy	628 (98.0)	308 (96.3)	320 (99.7)	
Unwanted pregnancy	13 (2.0)	12 (3.8)	1 (0.3)	
Planned pregnancy				.239
Yes	512 (79.8)	250 (77.9)	262 (81.6)	
No	130 (20.2)	71 (22.1)	59 (18.4)	
COC^c^				.253
< 50 %	463 (72.1)	238 (74.1)	225 (70.1)	
> =50 %	179 (27.9)	83 (25.9)	96 (29.9)	
Attending antenatal information courses				**.000**
Yes	238 (37.1)	71 (22.1)	167 (52.0)	
No	404 (62.9)	250 (77.9)	154 (48.0)	

^a^∑ incomes in the household/(1 + (x*0.5) + (y*0.3)) (x: number of adults living in the same household, y: number of children under the age of 18 years living in the same household [modified OECD scale] [23])

^b^ *n* = 641

^c^Continuity of Care index: $\mathrm{C}\mathrm{O}\mathrm{C}=\frac{{\displaystyle \sum {n}_j^2-n}}{n\left(n-1\right)}$ [26]

Bold values signify significant findings *P*<0.05

The majority of the women in the final data set werre aged between 21 and 35 years (82.2 %), werre co-habiting or married (94.1 %), employed (65.3 %), did not have tertiary education (58.6 %). 42.1 % did not have a foreign nationality (Table 2). Of the women, 42.1 % had a partner with tertiary education and 25.5 % had a partner with a foreign nationality.

**Table 2:** Study sample characteristics, chi-squared test reporting significance level for association with antenatal care utilisation, ordinal regression analysis reporting adjusted OR for being assigned into a higher CTP category

		Antenatal care utilisation classified by the CTP tool				*P* value	Adjusted OR
		Inadequate	Intermediate	Sufficient	Appropriate	χ^2^ test	
		(*N* = 49)	(*N* = 46)	(*N* = 214)	(*N* = 333)		
	Total (column %)	N (row %)	N (row %)	N (row %)	N (row %)		
Predisposing characteristics							
Age (years)						0.32(a)	(b)
≤20	8 (1.2)	0	0	6 (75.0)	2 (25.0)		
21–35	528 (82.2)	41 (7.8)	40 (7.6)	172 (32.6)	275 (52.1)		
>35	106 (16.5)	8 (7.5)	6 (5.7)	36 (34.0)	56 (52.8)		
Marital status						0.14(a)	(b)
Co-habiting or married	604 (94.1)	44 (7.3)	45 (7.5)	197 (32.6)	318 (52.6)		
Single	38 (5.9)	5 (13.2)	1 (2.6)	17 (44.7)	15 (39.5)		
Occupational status						**<0.001**	
Employed	419 (65.3)	20 (4.8)	26 (6.2)	120 (28.6)	253 (60.4)		
Unemployed	223 (34.7)	29 (13.0)	20 (9.0)	94 (42.2)	80 (35.9)		**0.49 (0.34-0.70)**
Educational level						**<0.001**	
Up to secondary	376 (58.6)	35 (9.3)	33 (8.8)	139 (37.0)	169 (44.9)		**0.60 (0.43-0.82)**
Tertiary	266 (41.4)	14 (5.3)	13 (4.9)	75 (28.2)	164 (61.7)		
Nationality						**0.009**	(b)
Belgian/Dutch	475 (74.0)	29 (6.1)	36 (7.6)	149 (31.4)	261 (54.9)		
All other nationalities	167 (26.0)	20 (12.0)	10 (6.0)	65 (38.9)	72 (43.1)		
Educational level partner						**<0.001**	(b)
No partner	38 (5.9)	5 (13.2)	1 (2.6)	17 (44.7)	15 (39.5)		
Up to secondary	334 (52.0)	33 (9.9)	30 (9.0)	120 (35.9)	151 (45.2)		
Tertiary	270 (42.1)	11 (4.1)	15 (5.6)	77 (28.5)	167 (61.9)		
Nationality of the partner						**0.003**	(b)
No partner	38 (5.9)	5 (13.2)	1 (2.6)	17 (44.7)	15 (39.5)		
Belgian/Dutch	441 (68.7)	27 (6.1)	29 (6.6)	133 (30.2)	252 (57.1)		
All other nationalities	163 (25.4)	17 (10.4)	16 (9.8)	64 (39.3)	66 (40.5)		
Region						**0.009**	
Brussels Metropolitan	321 (50.0)	31 (9.7)	26 (8.1)	118 (36.8)	146 (45.5)		0.90 (0.64-1.26)
Urban Dutch regions	321 (50.0)	18 (5.6)	20 (6.2)	96 (29.9)	187 (58.3)		
Enabling characteristics							
Equivalent income						**<0.001**	(b)
Low	112 (17.4)	17 (15.2)	9 (8.0)	51 (45.5)	35 (31.3)		
Moderate	451 (70.2)	29 (6.4)	33 (7.3)	141 (31.3)	248 (55.0)		
High	79 (12.3)	3 (3.8)	4 (5.1)	22 (27.8)	50 (63.3)		
Health insurance cover						**0.008**(a)	(b)
Yes	623 (97.0)	46 (7.4)	46 (7.4)	202 (32.4)	329 (52.8)		
No	19 (3.0)	3 (15.8)	0 (0.0)	12 (63.2)	4 (21.1)		
Additional health insurance						**<0.001**	(b)
Yes	431 (67.1)	24 (5.6)	29 (6.7)	130 (30.2)	248 (57.5)		
No	211 (32.9)	25 (11.8)	17 (8.1)	84 (39.8)	85 (40.3)		
Pregnancy-related characteristics							
Parity						**0.042**	(b)
Primiparae	284 (44.2)	19 (6.7)	16 (5.6)	84 (29.6)	165 (58.1)		
Multiparae	358 (55.8)	30 (8.4)	30 (8.4)	130 (36.3)	168 (46.9)		
Wish for pregnancy^d^						0.51(a)	(b)
Wanted pregnancy	628 (98.0)	49 (7.8)	44 (7.0)	210 (33.4)	325 (51.8)		
Unwanted pregnancy	13 (2.0)	0 (0.0)	2 (15.4)	4 (30.8)	7 (53.8)		
Planned pregnancy						**0.013**	(b)
Yes	512 (79.8)	35 (6.8)	34 (6.6)	161 (31.4)	282 (55.1)		
No	130 (20.2)	14 (10.8)	12 (9.2)	53 (40.8)	51 (39.2)		
COC^e^						**0.041**	
<50 %	463 (72.1)	42 (9.1)	39 (7.8)	158 (34.1)	227 (49.0)		**0.60 (0.42-0.84)**
≥50 %	179 (27.9)	7 (3.9)	10 (5.6)	56 (31.3)	106 (59.2)		
Attending antenatal information classes						**<0.001**	
Yes	238 (37.1)	11 (4.6)	7 (2.9)	72 (30.3)	148 (62.2)		
No	404 (62.9)	38 (9.4)	39 (9.7)	142 (35.1)	185 (45.8)		**0.67 (0.47-0.94)**

^a^The condition for the chi-squared test for larger contingency tables was not met: valid if less than 20 % of the expected numbers are under 5 and the minimum expected count is less than 1 ^37^

^b^Not included in the final model of ordinal logistic regression analysis

^c^∑ incomes in the household/(1 + (x*0.5) + (y*0.3)) (x: number of adults living in the same household, y: number of children under the age of 18 years living in the same household [modified OECD scale] [23]

^d^ *n* = 641

^e^Continuity of Care index: $\mathrm{C}\mathrm{O}\mathrm{C}=\frac{{\displaystyle \sum {n}_j^2-n}}{n\left(n-1\right)}$ [26]

Bold values signify significant findings *P*<0.05

With regard to the enabling characteristics, 70.2 % of the women had a moderate equivalent income, 97.0 % had health insurance cover and 32.9 % had no additional health insurance cover.

The pregnancy-related characteristics revealed that 55.8 % of the women were multiparae. Pregnancy was wanted for 98.0 % of the women but unplanned for 20.2 %. A lower continuity of care provider, represented by a COC index < 50 %, was observed for 72.1 % of the women, while 62.9 % did not attend antenatal information classes.

### Comparison of ANC utilisation between both regions

ANC utilisation differs significantly between regions (*p* = 0.009) (Tables 2 and 3). According to the classification by the CTP tool, 9.7 % of the women from the Belgian subsample had an inadequate care trajectory compared with 5.6 % in the Dutch subsample. Furthermore, only 45.5 % of the women in Belgium, compared to 58.3 % of Dutch women, were assigned to the appropriate ANC group (Table 3).

**Table 3:** Comparison of antenatal care utilization between regions (*N* = 642)

	Total	Brussels Metropolitan Region	Urban Dutch regions	*p*-value χ^2^ test
	(*N* = 642)	(*N* = 321)	(*N* = 321)	
	N (column %)	N (column %)	N (column %)	
Content and Timing of Pregnancy care				
Inadequate	49 (7.6)	31 (9.7)	18 (5.6)	**0.009**
Intermediate	46 (7.2)	26 (8.1)	20 (6.2)	
Sufficient	214 (33.3)	118 (36.8)	96 (29.9)	
Appropriate	333 (51.9)	146 (45.5)	187 (58.3)	

Bold values signify significant findings *P*<0.05

### Individual determinants of ANC utilisation

The predisposing characteristics of occupational status (*p* < 0.001), educational level and nationality of the women (*p* < 0.001; *p* = 0.009 respectively) and their partners (*p* < 0.001; *p* = 0.003 respectively) were found to be significantly associated with ANC utilisation (Table 2). Appropriate ANC use was higher among women with tertiary education (61.7 %), who were employed (60.4 %) and who were native (54.9 %) compared with women with secondary level education (44.9 %), who were unemployed (35.9 %) and had a foreign nationality (43.1 %) respectively.

Concerning the enabling characteristics, results showed that the higher the equivalent income, the higher the proportion of women with appropriate ANC utilisation (*p* < 0.001). More than half of the women with moderate (55.0 %) or high equivalent income (63.3 %) received appropriate ANC. This proportion was 31.3 % among women with low equivalent income. Women with health insurance and additional health insurance cover received appropriate content and timing of pregnancy care more often than women without this coverage (52.8 % versus 21.1 % and 57.5 % versus 40.3 % respectively) (*p* = 0.008 and *p* < 0.001 respectively).

With respect to pregnancy-related characteristics, appropriate care use was higher among primiparae (58.1 %), women with a planned pregnancy (55.1 %), women who had a COC index ≥ 50 % (59.2 %) and women who attended antenatal information classes (62.2 %) compared with multiparae (46.9 %), women with an unplanned pregnancy (39.2 %), women who had a COC index < 50 % and women who did not attend antenatal information classes (45.8 %) respectively (*p* < 0.05).

In the final model of the multivariate analysis, after adjustment for confounding variables (Table 2), the overall regional variable – the Belgian versus the Dutch subsamples – did not remain significantly associated with ANC use. However, four variables were significantly associated with ANC utilisation when controlling for the other variables. Women with no more than a secondary education (OR: 0.60; 95 % CI 0.43–0.82) and unemployed women (OR: 0.49; 95 % CI 0.34–0.70) had lower odds of being assigned to a higher CTP category compared with women with tertiary education and employment respectively.

In the final model no enabling characteristics remained significantly associated with the content and timing of ANC.

Women with a COC index < 50 % (OR: 0.60; 95 % CI 0.42–0.84) and women who did not attend antenatal information classes (OR: 0.67; 95 % CI 0.47–0.94) had lower odds of obtaining a higher CTP classification compared with women with a COC index ≥ 50 % and those attending antenatal information classes respectively.

## Discussion

This study compares ANC utilisation as classified by the CTP tool between two groups of ANC attendees in two different countries and identified predisposing, enabling and pregnancy-related determinants based on a pooled data set. To our knowledge this is the first international comparative study that has considered these three factors related to the content and timing of ANC. Unadjusted analysis reveal that women in urban Dutch regions receive more appropriate ANC than women in the Brussels Metropolitan Region. However, multivariate analysis do not indicate that the region in itself is a determinant of ANC utilisation when controlling for all individual characteristics. This finding makes the study unique. Irrespective of the region, adequate content and timing of ANC is associated with higher educational level, employed status, higher continuity of care and attendance of antenatal information classes.

Previous studies have shown that a low educational level is associated with late initiation of ANC [7, 9], a low number of antenatal visits [6, 28], receiving no care at all [6] and a lower probability of being in a higher CTP category [8]. Lack of a paid job [9] and type of occupation [29] have also been related to inadequate ANC use. Choté et al. suggested that education may influence ANC use due to the level of general health knowledge and health literacy [9]. The knowledge and skills acquired through education may create better access to information, stimulate receptiveness to health education messages and thus enable to access and communicate with health care providers [30].

The social network, which may be less extended in unemployed women might be a mechanism explaining the association of employment with ANC use. Information and encouragement received through a social network may stimulate women to use care [31, 32].

No enabling characteristics, such as income, was retained in our final model. The compulsory universal cover offered by health insurers, which includes basic ANC in both Belgium [13] and the Netherlands [33] may play a part. However, the provision of universal cover seems to be insufficient to offset disparities in ANC utilisation [29]. The use of health care services can be measured in terms of realised access to these services [4]. Inequitable access occurs when important structural aspects of society determine who receives appropriate ANC. However, a sole focus on measures designed to alter these aspects – such as educational level and employment status – for the sake of promoting equitable access, is hard due to their low mutability [4]. Other measures, such as the promotion of health literacy and knowledge from an early age through the education system or the training of health professionals in communication skills to adapt to the health literacy level of the care seeker, may encourage better utilisation of care [34].

With regard to pregnancy-related determinants, this study demonstrates that a lower continuity of ANC provider is associated with a lower CTP category. This index is calculated without differentiating between the type of primary caregiver – in Belgium most often an obstetrician and in the Netherlands a midwife. These results indicate that the continuity of care provider is important for the appropriateness of care irrespective of the type of provider. Attending antenatal classes is related to receiving more appropriate ANC, although the number and content of these classes were not considered. While non-attenders are not convinced that antenatal classes might benefit them, attenders consider them to be valuable [35]. Similarly, non-attenders may be less convinced of the importance of and need for ANC, which may hinder appropriate ANC use. Non-attenders of antenatal education classes are found to come from more vulnerable groups, with a low level of education or being unemployed [36]. Enhancing the awareness of the importance of appropriate follow-up and the advantages of antenatal classes may stimulate care use.

Cross-border data-sharing enabled the study of ANC utilisation in two countries. However there are some limitations to the study. The number of variables used in this study was restricted by the variables equally examined and operationalised in the original studies [8, 14]. For example, origin or ethnicity could not be examined in this study due to different operationalization of the variables in both datasets, although previous studies have identified these variables as important determinants of ANC use [2, 5, 6, 8, 9]. These differences in the data sets could lead to possible bias of the results. Furthermore, it would be valuable to extend the set of determinants with more elements of the health care system (eg main care provider, reimbursement system) to unravel their role in relation to antenatal care utilisation. In both studies only women that seek care were included. Therefore we are unable to draw conclusions in this specific group of women.

## Conclusions

While it could be expected that the country women live in, with a specific health care system, would have an impact on the appropriateness of antenatal care use, personal characteristics seemed to have a larger impact. The results of our study demonstrate that educational level and employment status are important factors in obtaining appropriate content and timing of ANC in both regions. One way to promote appropriate ANC and influence practice would be to introduce measures encouraging women to attend antenatal classes, for example by providing classes free of charge to socially vulnerable women. The organisation of public education about the (importance of) antenatal care is another recommendation for practice. Furthermore, it is important to systematically create maternal health care models in which the continuity of care provider is ensured. All are modifiable factors that will contribute to more appropriate care use and can be considered by perinatal health care practitioners.

This is the first study measuring received content and timing of care in pregnancy (CTP) across countries. Despite the value of this study, more cross-border studies are required including other/more countries with varying health care systems. A pan-European approach would be appropriate in order to perform collaborative research aiming at increasing the uptake of antenatal care. Further other individual determinants, such as origin, social network and health beliefs with regard to pregnancy and care could be examined. These future studies should also use a larger sample including women residing in both urban and non-urban regions. To achieve this, systematic and routine data collection that provides information on elements of the CTP tool and the individual characteristics of pregnant women will be required.

## Abbreviations

ANC, antenatal care; CTP-tool, content and timing of care in pregnancy tool
